# Hyperpolarized ^129^Xe imaging of the brain: Achievements and future challenges

**DOI:** 10.1002/mrm.29200

**Published:** 2022-03-07

**Authors:** Yurii Shepelytskyi, Vira Grynko, Madhwesha R. Rao, Tao Li, Martina Agostino, Jim M. Wild, Mitchell S. Albert

**Affiliations:** ^1^ Chemistry Department Lakehead University Thunder Bay Ontario Canada; ^2^ Thunder Bay Regional Health Research Institute Thunder Bay Ontario Canada; ^3^ Chemistry and Materials Science Program Lakehead University Thunder Bay Ontario Canada; ^4^ POLARIS, Unit of Academic Radiology, Department of IICD University of Sheffield Sheffield UK; ^5^ Insigneo Institute for in Silico Medicine Sheffield UK; ^6^ Northern Ontario School of Medicine Thunder Bay Ontario Canada

**Keywords:** brain, HP ^129^Xe brain imaging, hyperpolarized xenon‐129, magnetic resonance imaging/spectroscopy, perfusion imaging

## Abstract

Hyperpolarized (HP) xenon‐129 (^129^Xe) brain MRI is a promising imaging modality currently under extensive development. HP ^129^Xe is nontoxic, capable of dissolving in pulmonary blood, and is extremely sensitive to the local environment. After dissolution in the pulmonary blood, HP ^129^Xe travels with the blood flow to the brain and can be used for functional imaging such as perfusion imaging, hemodynamic response detection, and blood–brain barrier permeability assessment. HP ^129^Xe MRI imaging of the brain has been performed in animals, healthy human subjects, and in patients with Alzheimer's disease and stroke. In this review, the overall progress in the field of HP ^129^Xe brain imaging is discussed, along with various imaging approaches and pulse sequences used to optimize HP ^129^Xe brain MRI. In addition, current challenges and limitations of HP ^129^Xe brain imaging are discussed, as well as possible methods for their mitigation. Finally, potential pathways for further development are also discussed. HP ^129^Xe MRI of the brain has the potential to become a valuable novel perfusion imaging technique and has the potential to be used in the clinical setting in the future.

## 1 INTRODUCTION

There are multiple brain imaging modalities currently available for clinical diagnostic use, including ultrasound, CT, single‐photon emission CT, PET, and MRI. MRI is a noninvasive technique that uses no ionizing radiation and can produce images with high spatial resolution and contrast‐to‐noise ratio. Despite numerous developments and discoveries since MRI was invented in 1973,^1^ the main limitation of MRI remains the same: low sensitivity.^2^, ^3^ The MRI signal originates from the net magnetization of the sample due to the small population difference between the Zeeman energy levels of nuclei with typically a one‐half spin number. Conventional MRI uses the NMR signal from water protons (^1^H); numerous contrast agents are being developed to enhance the ^1^H MRI signal and provide the ability to localize the area of interest.^3^, ^4^, ^5^ Many of these agents, such as gadolinium chelated contrast agents, are focused on decreasing the spin–lattice (T_1_) and effective spin–spin (T_2_^*^) relaxation of ^1^H nuclei, which increases the MR contrast in T_1_‐weighted and T_2_^*^‐weighted images. Despite the wide use of ^1^H contrast agents, this approach is limited due to the presence of the background signal from surrounding tissues, which limits any increase in contrast‐to‐noise ratio. Additionally, there are a variety of techniques, such as BOLD functional MRI, arterial spin labeling (ASL), and MRA, which require multiple image acquisitions and complicated image postprocessing procedures for accurate data interpretation.^6^, ^7^

Another fundamentally different method for enhancing the MRI signal involves creating a hyperpolarized (HP) nuclear state.^8^ The HP state is a metastable state that can achieve up to a 10^5^ times larger spin population excess, compared with the thermal equilibrium state. Traditional HP MRI techniques work with non‐proton MRI‐sensitive nuclei such as xenon‐129 (^129^Xe), helium‐3 (^3^He), and carbon‐13 (^13^C).^9^, ^10^, ^11^, ^12^ The signal from HP nuclei can be enhanced by up to 10^5^ times, and MRI images of HP agents can be acquired with almost no background signal. Due to this signal boost, imaging of low‐concentration HP agents becomes possible. Currently, the main application of HP gas MRI is for lung imaging of healthy individuals and individuals with lung disorders.^2^, ^12^ HP ^129^Xe undergoes gas exchange in the lungs,^13^, ^14^, ^15^ easily dissolves in pulmonary blood,^12^, ^15^, ^16^ and then distributes throughout the body. Because HP ^129^Xe has a sufficiently long T_1_ relaxation time in the blood (T_1_ in a range of 3.4–7.8 s),^17^, ^18^, ^19^, ^20^ HP ^129^Xe MRI has the potential to produce functional images of highly perfused organs.^8^, ^21^, ^22^, ^23^ Although this idea was originally formulated at the end of the 20th century,^8^ HP ^129^Xe imaging in the brain is only recently under extensive development, and HP ^129^Xe imaging of the kidneys has just been demonstrated about a year ago.

Despite the intensive development of dissolved‐phase HP ^129^Xe imaging in brain tissues over the past decade, there have been no dedicated comprehensive reviews for the advances in this area. This review article aims to highlight the current progress and development in the field of HP ^129^Xe brain imaging, as well as discuss the technical challenges associated with this technology. The imaging approaches currently used are also reviewed and discussed. It is anticipated that insights into the challenges and opportunities of this field can be highlighted and aid in further advancements in the methodology and technique development of this technology with subsequent clinical translation.

## 2 HP ^129^XE SPECTROSCOPY AND CSI OF THE BRAIN

**FIGURE 1:**
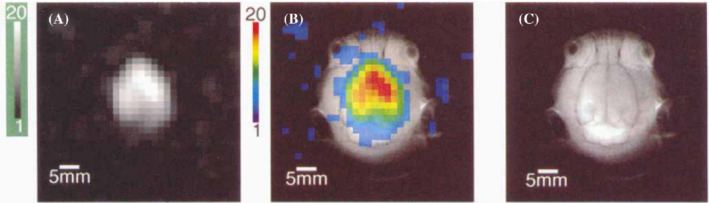
(A) Hyperpolarized (HP) xenon‐129 (^129^Xe) axial 2D CSI of the rat brain in grayscale. (B) Color‐coded overlay onto a high‐resolution proton image. The SNR of the HP ^129^Xe image was equal to 20. (C) High‐resolution proton spin‐echo MRI image used for brain localization. Images are reprinted with permission from the publisher^32^

Historically, xenon was used in medicine as an anesthetic due to its ability to dissolve in brain tissue.^24^, ^25^, ^26^, ^27^, ^28^ In addition to its anesthetic applications, xenon was widely used for cerebral blood flow evaluation using Xenon CT (Xe‐CT).^29^, ^30^, ^31^ Implementation of the hyperpolarization process for boosting the ^129^Xe MRI signal established an entirely new field of brain imaging and investigations with HP ^129^Xe.^8^ One of the properties that is most important for brain research with HP ^129^Xe dissolved in various brain tissues is its chemical shift. The first in vivo ^129^Xe brain MR spectrum was obtained by Swanson et al. in 1997 from the rat brain.^32^ A single blood‐tissue resonance peak was identified and used to produce an HP ^129^Xe 2D CSI of the rat brain (Figure 1). Later that year, Mugler et al. performed the first ^129^Xe MRS study of the human head.^33^ In that study, volunteers inhaled between 300 and 500 ml of HP ^129^Xe in one breath; 15 consecutive spectra of the head were subsequently acquired during and after a 15‐s breath‐hold period. The spectra showed one peak from the gas phase and one peak from the dissolved phase that was shifted 196 ppm from the gas peak. The dissolved phase peak appeared at the end of the inhalation period at approximately 5 s and disappeared 40 s after the start of the breath‐hold. The main limitation for the acquisition of human brain images at that time was the extremely low polarization of HP ^129^Xe achievable, approximately 2%.^33^

Obtaining a spectral peak from HP ^129^Xe dissolved in the brain allowed the conduction of a dynamic study of the distribution of xenon in the rat brain using 1D and 2D CSI.^34^ Swanson et al. detected a signal from the rat brain using 1D CSI with a low flip angle and investigated the time evolution of this signal. The polarization of ^129^Xe in this study, however, was still low (5%–8%); the study was performed primarily to observe the signal evolution within the body of the rat.

Duhamel et al. used a different approach for observation of the HP ^129^Xe solubility in the rat brain at 2.35 T.^35^ They injected naturally abundant HP ^129^Xe dissolved in a lipid emulsion, into the carotid artery, and observed two peaks at 199 and 194 ppm. These peaks were identified as ^129^Xe dissolved in the tissue and the intravascular compartment, respectively.^35^ It was clear that the signal intensity was too small to observe peaks from all brain tissues. Therefore, a final conclusion regarding the specific resonance frequencies of all ^129^Xe compartments, rather than merely just their frequency ranges, was not possible. Wakai et al. was able to observe all the individual ^129^Xe resonances by averaging 60 acquisitions during continuous breathing of an enriched HP ^129^Xe gas mixture. They observed five ^129^Xe spectral peaks in the rat brain that ranged between 189 and 210 ppm.^36^ Following this work, Nakamura et al. assigned a spectral peak at 195 ppm to the brain tissue: one at 210 ppm to HP ^129^Xe dissolved in the blood, and one at 189 ppm to non‐brain tissues (assumed to be muscle).^37^ Their conclusions on the resonance frequencies of HP ^129^Xe in the brain were aided by using a rat model involving an arterial ligation. Kershaw et al. found the peaks at 195 and 192 ppm originated from gray and white matter, respectively.^38^ Additionally, the peaks at 189 and 198 ppm were interpreted as signals from the jaw muscle and fat tissue.^38^

**FIGURE 2:**
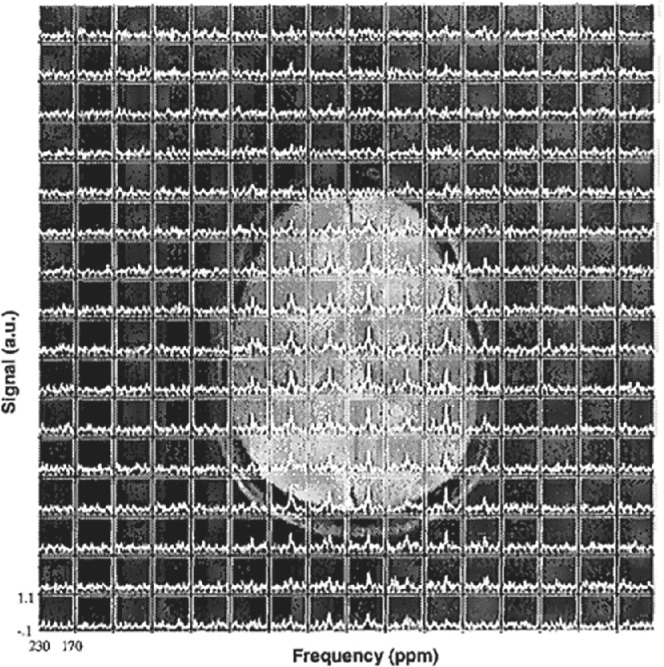
Two‐dimensional‐CSI spectra of HP ^129^Xe dissolved in brain tissue superimposed onto a ^1^H image. The image was reprinted with permission from the publisher^39^

The ability to distinguish the HP ^129^Xe peaks in the human brain has become possible with the availability of increased xenon polarization. 1D CSI spectra of the human brain with ^129^Xe polarized up to 8%, and a 2D‐CSI image using 14% polarized ^129^Xe dissolved in brain tissue superimposed on a ^1^H image, were obtained by Kilian et al. in 2002.^39^ Two additional peaks at 198 and 195 ppm were observed on the 1D‐CSI spectra beside an already identified peak at 196 ppm from Mugler et al.'s results.^33^ The 2D‐CSI measurements (Figure 2) revealed at least three additional peaks at 185, 193, and 200 ppm after spectral averaging, in addition to a peak previously observed at 197 ppm.^39^ Following their initial study, Kilian et al. performed an additional 2D CSI using isotopically enriched HP ^129^Xe to determine the origin of ^129^Xe peaks in the tissue compartment.^40^ The authors observed two dominant peaks from HP ^129^Xe in the brain region at 196 and 193 ppm, and two additional minor peaks from HP ^129^Xe in non‐brain tissues located below the brain at 190 and 201 ppm. The origins of the dominant peaks at 196 and 193 ppm were proposed to come from the gray and white matter, respectively.

**FIGURE 3:**
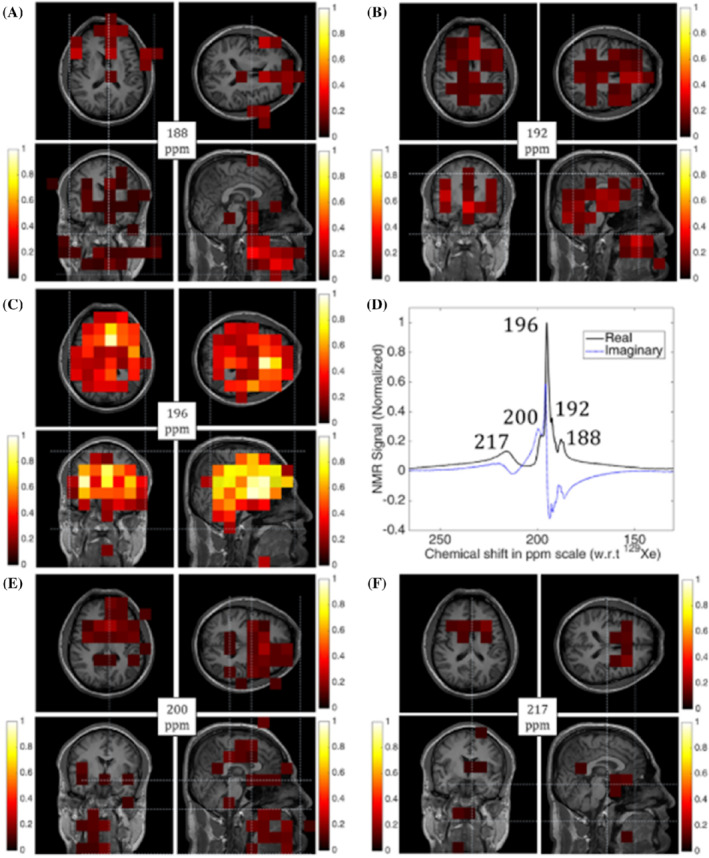
Two‐dimensional CSI of spatially resolved peaks from ^129^Xe in the human head superimposed onto ^1^H images. (A) Tissue in the cheek muscle and the midbrain/brainstem. (B) White matter and cartilaginous soft tissue. (C) Gray matter. (D) Spectra of the whole brain with a bandwidth of 136.0 9 ppm and a spectral resolution of 0.33 ppm. (E) Body interstitial fluid/plasma, fat tissue outside of the brain, and CSF. (F) Red blood cells (RBCs). The figure was reprinted with permission from the publisher^42^

After more than 10 years since these animal studies, during which time the polarization of HP ^129^Xe was significantly improved, Rao et al. demonstrated the first HP ^129^Xe human brain CSI, with detailed spectroscopy, at 1.5 T in 2015, where a red blood cell (RBC) ^129^Xe peak was observed for the first time.^41^ During the following year, they published a detailed study on the assignments of all the observed HP ^129^Xe brain peaks, which was based on high‐resolution spectroscopy and CSI measurements.^42^ In the latter study, 3 healthy volunteers each inhaled 1 L of HP ^129^Xe, followed by a 20‐s breath‐hold, during which the acquisition was performed. CSI was conducted to assign the HP ^129^Xe peaks obtained from the spectroscopy results (Figure 3D) to various tissue compartments within the head. An HP ^129^Xe peak at 188 ppm (Figure 3A) was assigned to HP ^129^Xe dissolved in soft muscular tissue in the cheek and ^129^Xe in the midbrain. The peak at 192 ppm (Figure 3B) corresponded to HP ^129^Xe dissolved in white matter; the peak at 196 ppm (Figure 3C) corresponded to HP ^129^Xe dissolved in gray matter; and the peak at 200 ppm (Figure 3E) was assigned to HP ^129^Xe dissolved in the plasma, fat tissue outside of the brain, and CSF. The final peak observed at 217 ppm (Figure 3F) showed high signal intensity at the location of the Circle of Willis and corresponded to HP ^129^Xe dissolved in RBCs. The results of this study mostly agreed with the results obtained from previous studies using animal models. One difference, however, was in frequency of the RBC peak, which was reported at 210 ppm in rats, and at 217 ppm in humans.

A spectroscopic study by Li et al^43^ evaluated the influence of the pulmonary oxygen concentration on the HP ^129^Xe brain signal. The authors proposed an “apparent relaxation time” as a parameter that reflects the dependence of the HP ^129^Xe brain signal on the pulmonary oxygen concentration. The optimal pulmonary oxygen concentration range for maximizing the SNR of ^129^Xe brain images was reported to be between 25% and 35%, which agrees with previous experimental and theoretical findings.^44^

Antonacci et al. raised an important question of the effect of the macroscopic susceptibility gradients on the dissolved‐phase HP ^129^Xe chemical shift.^45^ They pointed out the lack of consistency of the HP ^129^Xe chemical shift dissolved in the same tissues in the different studies. To solve this problem, they proposed a novel method for mitigation of the effects of the macroscopic susceptibility gradients by referencing the dissolved ^129^Xe resonances with the chemical shifts of the nearby ^1^H water protons. This allows the comparison of the chemical shift values from different studies and aids in the correct identification of the origin of the peaks.

## 3 RELAXATION TIME MEASUREMENTS

Other important characteristics of HP ^129^Xe are the spin–lattice or longitudinal (T_1_) and spin–spin or transverse (T_2_) relaxation times. The image quality depends on the TR and TE, which are set based on the longitudinal and transverse relaxation time values, respectively. The first measurement of the longitudinal relaxation was performed ex vivo in rat brain tissue at 9.4 T by Wilson et al.^46^ T_1_ relaxation times were determined at varying oxygenation levels and were reported to be 18 ± 1 s in the oxygenated state and 22 ± 2 s in the deoxygenated state. Following this ex vivo study, Duhamel et al. measured the longitudinal and transverse magnetization in vivo at 2.35 T.^47^ The T_1_ of HP ^129^Xe dissolved in brain tissue was calculated to be 14 ± 1 s, and the T_2_^*^ was measured to be 8.0 ± 1.2 ms.^47^ Spin–lattice relaxation for the white matter was also derived from human brain dynamic spectra by Kilian et al. to be 8 s.^48^ The next measurement of T_1_ relaxation was performed in vivo in the rat brain by Wakai et al, who proposed a method for measuring the longitudinal relaxation without the need for an estimation of the flip angle.^49^ Using this approach, the longitudinal relaxation time of ^129^Xe in the rat brain was found to be 26 ± 4 s. Due to the large discrepancies reported for T_1_ in these studies, in 2008, Zhou et al. reinvestigated the longitudinal relaxation time of ^129^Xe dissolved in the rat brain by developing a mathematical description of the HP ^129^Xe wash‐out process from the brain.^50^ The authors determined the longitudinal relaxation time of ^129^Xe dissolved in the rat brain using a two‐pulse method (T_1_ = 15.3 ± 1.2 s) and a multipulse protocol (T_1_ = 16.2 ± 0.9 s).^50^

The effective spin–spin relaxation (T_2_^*^) for the ^129^Xe gray‐matter peak in the rat brain was estimated primarily from the linewidth at half‐height of the peak by Mazzanti et al. to be 5.42 ± 0.3 ms at 4.7 T (observed at 194.7 ppm),^51^ and by Rao et al. in the human brain at 1.5 T to be 8.8 ms.^42^

In summary, there is a lack of consistency among the measured relaxation values (Table 1). Furthermore, there were no T_1_ measurements performed for HP ^129^Xe dissolved in the gray matter in humans. An accurate assessment of HP ^129^Xe T_1_ in the gray matter at different magnetic field strengths is vital for the practical implementation of HP ^129^Xe brain imaging. Indeed, one of the main potential applications of HP ^129^Xe brain imaging is rapid quantification of cerebral perfusion, as well as blood–brain barrier permeability. According to multiple mathematical models that were developed,^48^, ^52^, ^53^, ^54^ however, the HP ^129^Xe signal dynamics depend equally on both tissue perfusion and the T_1_ relaxation time. Therefore, until T_1_ is quantified with high accuracy in healthy individuals as well as in patients with neurodegenerative or cerebrovascular diseases, brain perfusion imaging using HP ^129^Xe MRI will remain mostly qualitative.

**TABLE 1:** Chemical shift, longitudinal, and transverse relaxation of HP ^129^Xe dissolved in the different brain tissues

	Chemical shift relative to gas peak (ppm)		T_1_ (s)	T_2_^*^ (ms)
Rat brain studies	Tissue	194.5 (2.0 T)^32^ 199 (2.35 T)^35^ 195 (4.7 T)^37^ 198 (4.7 T)^38^	14.0 ± 1.0 (2.35 T)^47^ 3.6 ± 2.1 (2.35 T)^55^ 26 ± 4 (4.7 T)^49^ 15.3–16.2 (4.7 T)^50^	8.0 ± 1.2 (2.35 T)^47^ 5.42 ± 0.3 (4.7 T)^51^
	Muscle	189 (4.7 T)^37^ 189 (4.7 T)^38^		
	Blood	210 (4.7 T)^37^		
	Gray matter	195 (4.7 T)^38^		
	White matter	192 (4.7 T)^38^		
Arterial blood			18 ± 1 (oxygenated 9.4 T)^46^ 22 ± 2 (deoxygenated 9.4 T)^46^ 13.7 ± 1.6 (4.7 T)^17^ 13–16 (1.5 T)^19^	
Human brain studies	Gray matter	196.5 (2.94 T)^48^ 196 (3 T)^40^ 196 (1.5 T)^42^		8.8 (1.5 T)^42^
	White matter	193 (3 T)^40^ 192 (1.5 T)^42^	8 (2.94 T)^48^	
	RBCs	222 (1.5 T)^19^ 224 (4.7 T)^17^ 217 (1.5 T)^42^		
	Blood plasma	197 (1.5 T)^19^ 198 (4.7 T)^17^ 200 (1.5 T)^42^		
	Muscle tissue	188 (1.5 T)^42^		

Surprisingly, there were no T_2_^*^ measurements conducted for HP ^129^Xe dissolved in human brain white matter, cerebral blood plasma and RBCs, and soft muscle tissue. These measurements are essential for further HP ^129^Xe brain imaging pulse‐sequence development because they will allow proper optimization of the imaging TEs.

## 4 STRUCTURAL BRAIN IMAGING WITH HP ^129^XE

**FIGURE 4:**
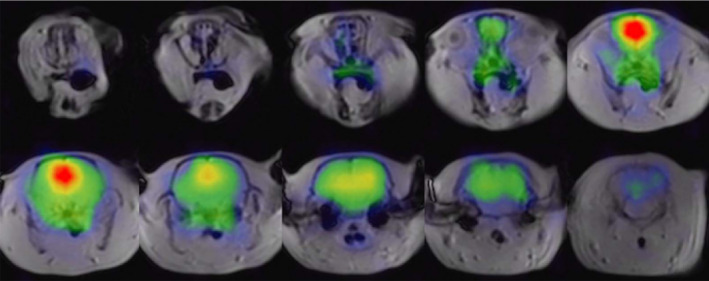
Three‐dimensional HP ^129^Xe MR images of rat brains. The dissolved HP ^129^Xe image (color) is overlaid onto a ^1^H anatomical image (grayscale). The ^129^Xe signal largely matches the brain tissue. The ^129^Xe signal was notably intense in the olfactory bulb and midbrain regions and was largely absent from the cerebellum. The images are reprinted with permission from the publisher^56^

At the end of the 20th century, the initial discovery of boosting the ^129^Xe signal with hyperpolarization for structural imaging of the brain using HP ^129^Xe was extremely promising.^8^ After signal‐intensity measurements at the beginning of the 21st century, however, it became clear that the polarization of HP ^129^Xe needed to be significantly higher than what was possible at that time. Swanson et al. measured an SNR of 20 in the rat brain with 3.1 × 3.1 × 10 mm^3^ voxels.^32^ Due to the limited concentration of HP ^129^Xe dissolved in brain tissue, CSI was used as the main imaging approach for HP ^129^Xe brain studies. To address the limitation of relying on CSI for HP ^129^Xe brain imaging, Nouls et al. developed a fast 3D radial gradient‐echo (GRE) acquisition approach for HP ^129^Xe brain imaging.^56^ They acquired high‐resolution 3D images of the HP ^129^Xe distribution in the rat brain with an isotropic 32 × 32 × 32 matrix and a voxel size of 3.65 × 3.65 × 3.65 mm^3^ (Figure 4).

Recently, Friedlander et al. demonstrated spectrally resolved HP ^129^Xe imaging of the rat brain using iterative decomposition with echo asymmetry and least‐square estimation (IDEAL) using a spiral readout at 3.0 T.^57^, ^58^ Using this approach, images of ^129^Xe dissolved in brain tissue and RBCs were acquired with an SNR of 31 ± 4 and 16 ± 2, respectively, and a resolution of 0.5 × 0.5 cm^2^.^57^ Using time‐resolved dynamic spiral IDEAL imaging, Friedlander et al. was able to perform, for the first time, ^129^Xe local blood–brain barrier (BBB) permeability assessment in hypercapnic and normocapnic rats during continuous breathing of HP ^129^Xe.^58^ Successful IDEAL decomposition of the dissolved‐phase HP ^129^Xe signal will likely be of interest for human brain imaging in future studies.

The first HP ^129^Xe structural human‐brain image was acquired at 1.5 T by Rao et al. in 2015, once the process of polarization was improved.^41^ The image was acquired in an axial projection using a 2D spoiled GRE sequence with a voxel size of 6.88 × 6.88 × 50 mm^3^. The HP ^129^Xe brain image correlated with the corresponding anatomical ^1^H MR image.

Rao et al. recently went on to perform 3D isotropic spectroscopic imaging of HP ^129^Xe in the human brain.^59^ The acquisition matrix was 10 × 10 × 10, yielding a slice thickness of 2 cm and an acquisition voxel size of 8 cm^3^. The acquired images were interpolated to a voxel size of 0.24 cm^3^ and a slice thickness of 0.625 cm. This novel approach for HP ^129^Xe spectroscopic imaging cold be potentially implemented in further brain oxygenation mapping.

**FIGURE 5:**
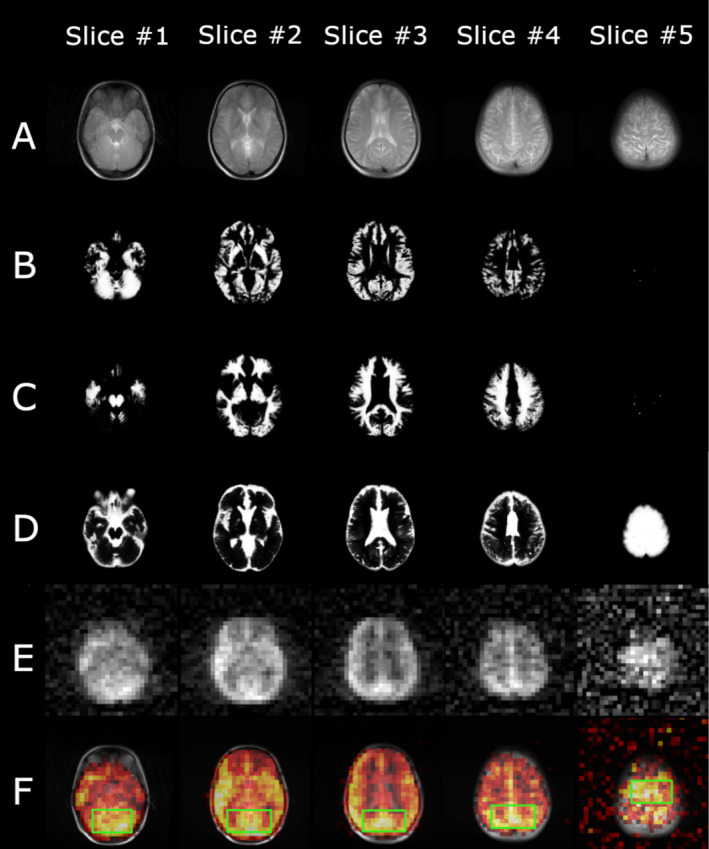
The first HP ^129^Xe 3D gradient‐echo (GRE) multislice image of the human brain. (A) ^1^H T_2_‐weighted anatomical axial turbo spin‐echo (TSE) images of a representative healthy volunteer. B‐D, Axial anatomical images of gray matter (B), white matter (C), and CSF (D) segmented using high‐resolution TSE ^1^H T_2_‐weighted images of a representative healthy volunteer. (E) Three‐dimensional GRE HP ^129^Xe axial brain slices acquired 10 s into the breath‐hold. (F) Thresholded HP ^129^Xe axial brain slice images superimposed on top of the corresponding ^1^H anatomical images from (A). It can be seen that the HP ^129^Xe signal corresponds well to the gray‐matter distribution in the brain. In addition, a partial correlation has been observed between the white‐matter images and the HP ^129^Xe images. The image was reprinted with permission from the publisher^60^

The most recent contribution to HP ^129^Xe structural brain imaging was achieved by Grynko et al. by acquiring 3D multislice images of the human brain at 3 T (Figure 5).^60^ Five slices of the human brain were imaged with a slice thickness of 20 mm and an acquisition voxel volume of 1.22 cm^3^, which is the smallest acquisition voxel volume of HP ^129^Xe human‐brain imaging currently achieved. The highest SNR was reported to be 18.76 ± 4.95 from the inhalation of 1 L of HP ^129^Xe polarized to about 50%.

These two recent studies provided a significant step forward, demonstrating the ability of HP ^129^Xe brain imaging to produce multiple slices, which will allow the accurate and precise anatomical localization of HP ^129^Xe dissolved in the human brain.

Despite these recent achievements in structural HP ^129^Xe imaging of the brain, the imaging voxel size remains approximately two orders in magnitude larger compared to that of conventional anatomical proton MRI (^1^H voxel size ∼ 10 mm^3^). The low concentration of ^129^Xe dissolved in the brain tends to significantly restrict the spatial resolution achievable for imaging. Based on previous uptake models^48^, ^61^ (detailed in the next section) and the Oswald solubility of ^129^Xe in pulmonary blood,^62^ it is estimated that only about 1%–2% of the amount of inhaled ^129^Xe actually dissolves in the brain tissues. In spite of the low concentrations of ^129^Xe achievable in brain tissue, however, it will be seen in the following sections that there is great value for using HP ^129^Xe for perfusion and other functional studies of the brain.

## 5 HP ^129^XE UPTAKE MODELS

The dynamics of HP ^129^Xe uptake in the brain and its wash‐out are complex and require careful consideration of multiple factors. Therefore, an accurate mathematical model of HP ^129^Xe signal dynamics is required for appropriate experimental design.

The first attempt to model ^129^Xe uptake in brain tissues was performed by Peled et al. in 1996.^61^ They proposed an uptake model that calculated the time‐dependent build‐up of polarized ^129^Xe in brain‐tissue compartments based on estimates of the ^129^Xe relaxation times in tissue, perfusion rates, arterial transmit time, and partition coefficients. The authors considered continuous breathing a dose of 70% enriched HP ^129^Xe mixed with 30% O_2_. The model predicted a maximum concentration of ^129^Xe of 27 uM for gray matter and 8 uM for white matter and myelin, reached at 60 s after inhalation. Martin et al. extended Peled's model by accounting for different breathing protocols and estimated the ^129^Xe concentration in the brain for a wide range of T_1_ values for the gas and tissue phases.^63^ The key ^129^Xe T_1_ parameters used in the model were as follows: 1000 s in the polarizer cell, 6 s in the arterial blood and in the tissue, and 12 s in the mouth and lungs. In this model, the polarization of ^129^Xe was assumed to be equal to 100%. The lung to brain transit time was estimated to be 5 s. Three different breathing protocols were investigated: continuous breathing, hyperventilation followed by a breath‐hold, and hyperventilation followed by continuous breathing. The maximum HP ^129^Xe concentration in the gray matter was calculated to be 0.09 mM at 15 s following inhalation for both the hyperventilation with a breath‐hold, and the continuous breathing methods. The ^129^Xe concentration in the brain was predicted to be 0.04 mM at 50 s after inhalation for the continuous breathing protocol.^63^ Although both of these initially proposed models did not account for such factors as chemical shift effects, HP ^129^Xe passage through biological membranes, and ^129^Xe exchange between brain compartments, they provided useful information for conducting further research. Kilian et al. went on to propose an improved ^129^Xe uptake model based on spectroscopic MR measurements at various time points after ^129^Xe inhalation.^48^ The model was in agreement with the quantitative ^129^Xe spectroscopy experimental data. Kilian also reported that the longitudinal ^129^Xe relaxation in gray matter was slower than that in white matter (T_1g_ > T_1w_).^48^ This model, however, also had drawbacks. It did not account for the gradient of ^129^Xe solubility in gray matter and white matter or the exchange of ^129^Xe between the tissues and the bloodstream. Following this, Shepelytskyi et al. expanded on Kilian's model for the case of HP ^129^Xe dynamic imaging, and implemented it for perfusion imaging of the human brain.^52^

An additional mathematical description of the HP ^129^Xe wash‐out process was developed by Zhou et al. for better estimation of the longitudinal relaxation time of ^129^Xe dissolved in the brain tissues.^50^ Following this, Kimura et al. developed an HP ^129^Xe uptake model and investigated ^129^Xe uptake using chemical shift saturation recovery spectroscopy in the mouse brain.^64^ This model allowed improvements to the estimation accuracy of the HP ^129^Xe longitudinal relaxation time in the mouse brain. Imai et al. also developed a theoretical model of the ^129^Xe signal dynamics in the mouse brain, and suggested a method for its quantitative measurement under continuous ^129^Xe ventilation conditions.^65^

The most recent kinetic model of HP ^129^Xe uptake was developed by Rao et al. for the determination of the transfer rate of inhaled xenon from cerebral blood to the gray matter in the human brain.^53^, ^66^ Using the time course of the HP ^129^Xe spectroscopic signal, the authors introduced a tracer kinetic model that explains the exchange of ^129^Xe between these compartments.^53^ In this model, the transient ratio of the HP ^129^Xe concentration from gray matter to the blood was calculated from single‐voxel MRS spectra. The slope of the transient ratio over time was proposed as a physiological marker of BBB permeability. The main advantage of Rao et al.'s model compared with other uptake models is that it considers the forward exchange of ^129^Xe between the cerebral blood and gray‐matter tissue. However, it only considers the forward transfer of HP ^129^Xe, and neglects the gradient of the HP ^129^Xe concentration in the gray matter.

Further development of more complex mathematical models is required to accurately describe the concentration of HP ^129^Xe in the brain, and consequently, its signal dynamics. Future models must also make a special effort to describe the HP ^129^Xe diffusion processes in brain tissues. They must consider both forward and retrograde transfer of HP ^129^Xe as well as properly describe the HP ^129^Xe concentration gradient within the brain tissues. The diffusion coefficients of HP ^129^Xe in the cerebral blood and brain tissues currently remains unknown, which reduces the accuracy of the previously established models, as they often use the value of the ^129^Xe diffusion coefficient in aqueous solutions as a substitute for that in the brain. Dedicated measurements of these important physical parameters for HP ^129^Xe are required to make further progress on HP ^129^Xe uptake modeling.

## 6 PERFUSION IMAGING WITH HP ^129^XE

**FIGURE 6:**
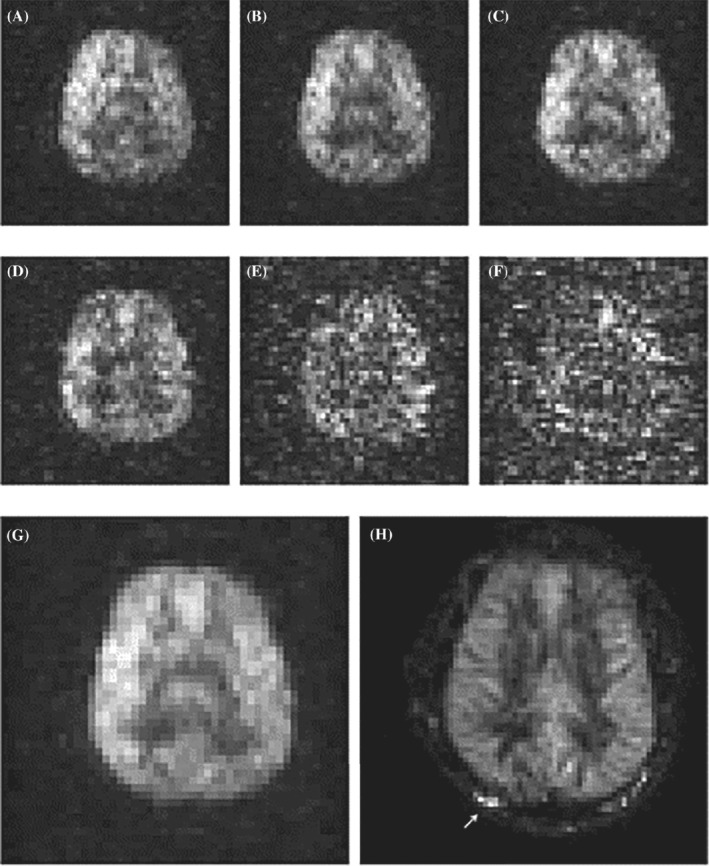
Brain perfusion in vivo images of a healthy volunteer. (A–F) HP ^129^Xe imaging at 1.5 T at 8 s (A), 16 s (B), and 24 s (C) after inhalation during a breath‐hold, and 32 s (D), 40 s (E), 48 s (F) after continuing breathing. (G) Average of the first four images (A–D) with 33‐s total imaging time. (H) Pseudo‐continuous arterial spin‐labeling (ASL) image at 3 T; summation of seven contiguous sections with total imaging time of 10 min. Images are reprinted with permission from the publisher^68^

Perfusion imaging is used widely in the clinic for the assessment of cerebrovascular physiology, to diagnose brain pathologies such as brain tumors and stroke. Hyperpolarized ^129^Xe is an exogenous agent that can act as an imaging agent for the evaluation of brain perfusion. This application was first predicted by Duhamel et al. in 2002, but the low polarization values of HP ^129^Xe (18%–20%) at that time did not permit the researchers to obtain high‐quality results for perfusion assessment in the rat brain.^47^ The next attempt for perfusion evaluation was performed by Rao et al. in the human brain in 2018, with HP ^129^Xe polarized to 35% at 1.5 T.^66^, ^67^, ^68^ Three healthy volunteers inhaled 1 L of 87% enriched ^129^Xe for a 24‐s breathhold duration. The HP ^129^Xe uptake images were acquired with time intervals of 8, 16, 24, 32, 40, and 48 after inhalation (Figure 6A–F). The volunteers resumed breathing at 24 s. The resulting images were zero‐padded up to an 80 × 80 in‐plane resolution from 7.81 × 7.81 × 130 mm^3^ voxels with a 32 × 32 resolution. The first four images were signal averaged for further comparison to ASL imaging. The signal averaged HP ^129^Xe images exhibited certain advantages over ASL perfusion imaging in that they did not require averaging over a period of several minutes, and they further lacked any undesired signals from blood vessels (Figure 6H). The SNR from the averaged images (Figure 6G) was 31 ± 9, 24 ± 4, and 23 ± 2 from the 3 healthy volunteers.^68^ Although this technique is limited by the quantity of ^129^Xe that is delivered to the brain and also by the loss of ^129^Xe polarization during transport from the lungs to the brain, it can be used for qualitative perfusion estimates in the human brain.

Recently, Rao et al. demonstrated a moderate correlation between the cerebral perfusion values as measured by ASL and the ^129^Xe uptake in the human brain.^69^ To investigate this correlation, the ASL perfusion images were corrected for any depolarization that ^129^Xe would experience using two exponential terms to account for the T_1_ polarization decay of HP ^129^Xe in the blood and during its residency time in gray matter. The authors reported a moderate positive correlation (correlation coefficient range of 0.34–0.63) between the corrected perfusion images obtained using ASL and the HP ^129^Xe brain images.

**FIGURE 7:**
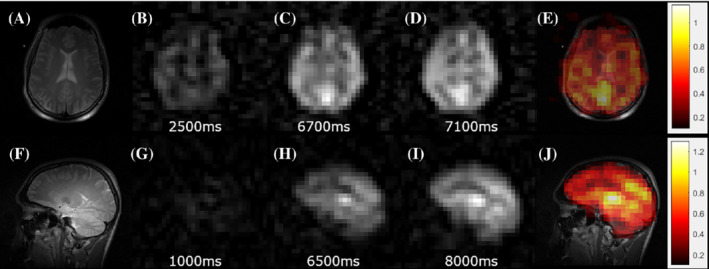
Example of perfusion map acquisition. (A,F) High‐resolution, T_2_‐weighted ^1^H scans for brain localization. (B–D) Three dynamic HP ^129^Xe time‐of‐flight (TOF) images acquired 2.5, 6.8, and 7.1 s after the application of a depolarization RF pulse in the axial projection. The gradual SNR increase can be observed with increasing wash‐in time. (E) The perfusion map created by the pixel‐by‐pixel recalculation of the TOF slope was used to calculate the sum of the perfusion rates of gray matter and white matter superimposed on top of a high‐resolution proton brain image. (G–I) Three dynamic TOF images acquired after 1, 6.5, and 8 s in the sagittal view. (J) Perfusion map in the sagittal view. Similar to (E), the intensity values were the net sum of the white‐matter and gray‐matter perfusion rates. Images are reprinted with permission from the publisher^52^

The most recent study on HP ^129^Xe perfusion imaging was conducted by Shepelytskyi et al. in 2020.^52^ They demonstrated a novel ^129^Xe time‐of‐flight (TOF) MRI technique capable of quantitative perfusion measurements. It was a different approach compared with that used by Rao et al.^68^ It was based on the time‐resolved depolarization of dissolved HP ^129^Xe in the brain and the acquisition of dynamic images after subsequent TOF wash‐in delays. It fostered the absence of any background signal and isolated the HP ^129^Xe delivered by the cerebral blood flow. Cerebral perfusion was recalculated from the dynamic HP ^129^Xe TOF images using a modified version of Kilian's HP ^129^Xe uptake model.^48^ Three dynamic TOF images were acquired using incremental TOF delay times: 2.5, 6.7, and 7.1 s for axial projections, and 1, 6.5, and 7.1 s for sagittal projections. The images were acquired using a 20 × 20 acquisition matrix with a 12.5 × 12.5 × 70 mm^3^ voxel volume, and zero‐padded to a 32 × 32 matrix. Figure 7 shows the dynamic HP ^129^Xe images and resulting perfusion map, overlaid on T_2_‐weighted ^1^H brain images.

In spite of the fact that several major advances have been made in the development of HP ^129^Xe perfusion imaging, this methodology remains largely in its infancy. There are numerous technical challenges associated with these approaches that originate from the issues highlighted previously. Most applications of HP ^129^Xe MRI for cerebral perfusion imaging remain, to a large extent, qualitative due to the fact that there have not been any imaging techniques developed that allow an accurate implementation of the existing HP ^129^Xe dynamic models. Shepelytskyi et al. carried out a quantitative estimation of cerebral perfusion^52^; however, they used several significant simplifications that somewhat reduce the accuracy of their estimations.

## 7 FUNCTIONAL MRI WITH HP ^129^XE

**FIGURE 8:**
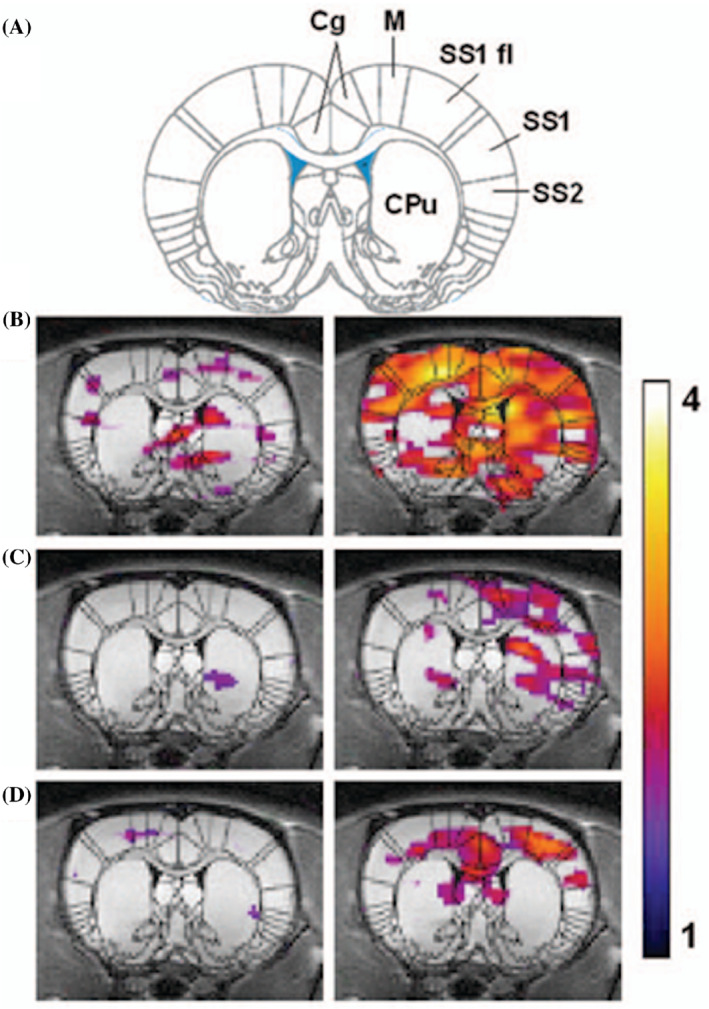
HP ^129^Xe fMRI data from three animals. The HP ^129^Xe signal is shown as a false‐color overlay on the corresponding 1‐mm‐thick coronal proton reference image taken from the same animal. The left panel shows the HP ^129^Xe signal intensity during baseline, and the right panel shows HP ^129^Xe signal intensity after injection of capsaicin 20 ul (3 mg/ml) into the right forepaw. The color scale represents SNR, and only signal with SNR above two are shown. Superimposition of a rat brain atlas (18) shows specific areas of the brain: cingulate cortex (Cg), the motor cortex (M), primary somatosensory cortex, SS1 forelimb region (SS1 and SS1 fl), the secondary somatosensory cortex (SS2), and striatum (CPu). The images were reprinted with permission from the publisher^51^

Because HP ^129^Xe acts as a natural cerebral blood flow tracer, it was suggested that HP ^129^Xe brain imaging should be capable of detecting physiological activity in the brain via changes in the local hyperpolarized ^129^Xe density contrast.^8^ The fundamental principles behind this mechanism are quite simple compared with the conventional BOLD technique for functional brain MRI (fMRI): Blood flow to areas of the brain that respond to stimulation is higher, and consequently, the local concentration of HP ^129^Xe in these regions will also be higher. Brain activation maps can therefore be created after subtraction of an HP ^129^Xe reference image acquired during a resting state. This approach was used by Mazzanti et al. in 2011, who first demonstrated the ability of HP ^129^Xe brain MRI to detect and map sensory stimulation of the rat brain.^51^ Two‐dimensional HP ^129^Xe CSI images were acquired before and after stimulation (Figure 8B–D) from injection of capsaicin into the fore‐paw. The authors observed an increase of the HP ^129^Xe signal in the somatosensory brain regions responsible for pain processing.^51^

**FIGURE 9:**
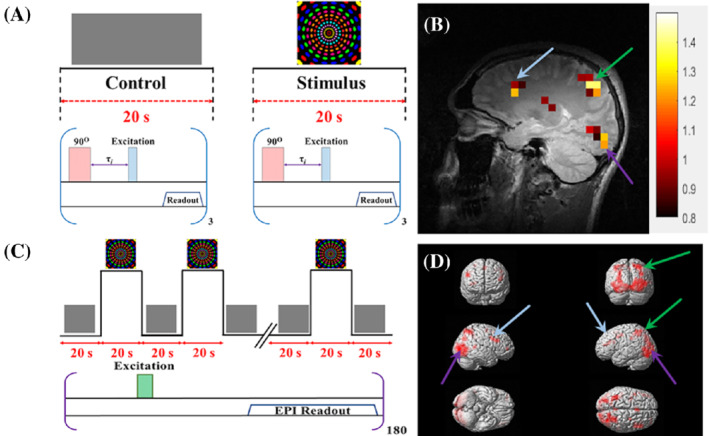
Detection of a hemodynamic response from a colorful visual stimulus using HP ^129^Xe perfusion mapping validated by BOLD brain functional MRI (fMRI). (A) Experimental design used for hemodynamic response detection. Two separate perfusion maps were acquired during the control (gray screen) and visual stimulation. (B) Hemodynamic response map created by subtracting the control perfusion map from the stimulated perfusion map and overlaid on top of a high‐resolution proton scan. Activation of the occipital lobe, superior parietal lobe, and frontal gyrus was observed. (C) BOLD fMRI experimental design for validation of the HP ^129^Xe technique. (D) BOLD fMRI 3D activation maps demonstrate a correlation with a ^129^Xe hemodynamic response map. The activated areas are indicated by colored arrows. Images are reprinted with permission from the publisher^52^

Although these early results seemed promising, this methodology was fraught with inherent errors and limitations. The direct subtraction approach for two HP ^129^Xe brain images is associated with a high level of potential errors caused by the interbreath‐hold variability of the HP ^129^Xe signal. This signal variability has been estimated to be about 30%, which can cause a large potential for false‐positive and false‐negative results during hemodynamic response mapping. This challenge was overcome by Shepelytskyi et al., who performed fMRI of the human brain using the novel HP ^129^Xe TOF imaging technique, which had been developed for perfusion assessment.^52^ The HP ^129^Xe TOF pulse sequence substantially reduced the interbreath‐hold signal variability^70^ and functioned well for an accurate assessment of the hemodynamic response (HDR).^52^ The HDR to visual and motor stimuli (Figure 9) was investigated. The resulting functional brain HDR maps (Figure 9B) correlated well with conventional ^1^H‐BOLD fMRI (Figure 9D).

Although a spatial correlation between the HP ^129^Xe HDR maps and conventional ^1^H‐fMRI images was observed, the HP ^129^Xe HDR maps had a substantially lower spatial resolution. The HDR maps had a single slice thickness of about 100 mm and an in‐plane pixel size of 7.81 mm^2^, whereas the conventional proton fMRI images were acquired with a 4‐mm slice thickness and 3.91‐mm^2^ in‐plane spatial resolution. Despite the significant limitations in spatial resolution, HP ^129^Xe HDR mapping outperformed conventional fMRI in terms of temporal resolution, as the whole brain was mapped in less than in 20 s.

## 8 BRAIN DISEASE DETECTION WITH HP ^129^XE

**FIGURE 10:**
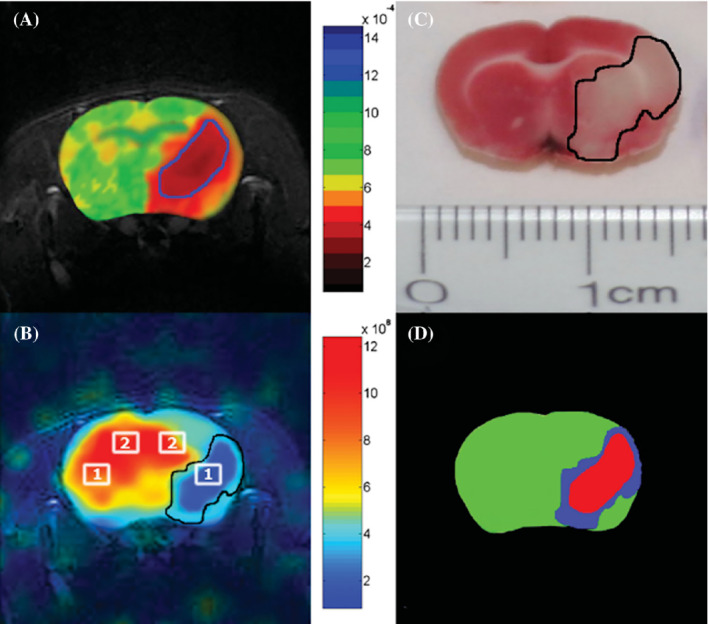
In vivo evaluation of stroke using 2D ^129^Xe CSI. (A) Representative ^1^H apparent diffusion coefficient map image obtained after a right middle cerebral artery occlusion. (B) Corresponding HP ^129^Xe 2D CSI indicating the large signal void corresponding to the ipsilesional hemisphere. (C) Corresponding 2,3,5‐triphenyltetrazolium chloride (TTC)–stained brain section of the same animal. (D) Tricolor map based on the ADC and TTC images shown in (A) and (C). Green, red, and blue represent nonischemic stroke. The images were reprinted with permission from the publisher^71^

Despite the low signal intensity of HP ^129^Xe dissolved in the human brain, it is possible to evaluate various differences in xenon physical properties between healthy subjects and subjects with brain‐related diseases. Zhou et al. demonstrated the first application of HP ^129^Xe brain CSI for in vivo ischemic stroke imaging^71^ in a rat model. The large hypointense region corresponding to the ischemic core (Figure 10C) was observed in an HP ^129^Xe image (Figure 10B).^71^ These results were corroborated by conventional ^1^H DWI as well as by histology (Figure 10A).

**FIGURE 11:**
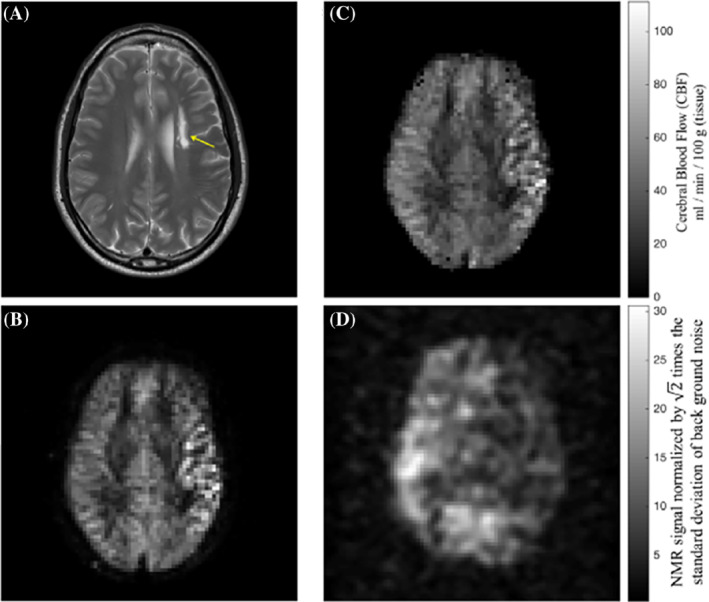
Brain MR images acquired in the same session from a subject with established stroke. (A) Axial T_1_‐weighted image showing infarct in the centrum semiovale of the left cerebral hemisphere (arrow). (B) An axial image from pseudo‐continuous ASL shows hyperintensity in the cerebral cortex adjacent to infarction. (C) Map of CBF estimated from ASL in (B) shows increased perfusion. (D) Hyperpolarized ^129^Xe brain image shows reduced uptake in the brain tissue supplied by the left internal carotid artery. The ^129^Xe signal in the region of hypointensity in (D) was 60% lower when compared with the average signal in the healthy region. Images are reprinted with permission from the publisher^72^

Following this initial study in animal models, Rao et al. conducted HP ^129^Xe brain perfusion imaging in a 52‐year old volunteer who had a stroke 2 years and 3 months before imaging with HP ^129^Xe.^72^ The conventional proton MRI revealed intracranial arterial occlusion with collateralization (Figure 11A). To evaluate perfusion using HP ^129^Xe, three 32 × 32 images were acquired during a breath‐hold at 8, 16, and 24 s after inhalation of 1 L of HP ^129^Xe with 35% polarization. The images were reconstructed up to an 80 × 80 in‐plane resolution with subsequent averaging from a 32 × 32 matrix with voxel size 6.875 × 6.875 × 50 mm^3^. The final image (Figure 11D) revealed a region of signal hypointensity, which indicated poor ^129^Xe uptake in the stroke area. The regional cerebral blood flow (Figure 11C) calculated from pseudo‐continuous ASL (Figure 11B), however, was higher in the same area, which indicated a delayed hyperperfusion. The lower ^129^Xe signal can be explained by a shorter mean transit time due to a higher cerebral blood flow. This reduces the transfer of ^129^Xe to the tissue and delays the delivery of ^129^Xe to that area, which affects the magnetization because of its T_1_ decay. Overall, this pioneering study demonstrated proof‐of‐principle contrast for using HP ^129^Xe imaging for stroke imaging in human subjects.

**FIGURE 12:**
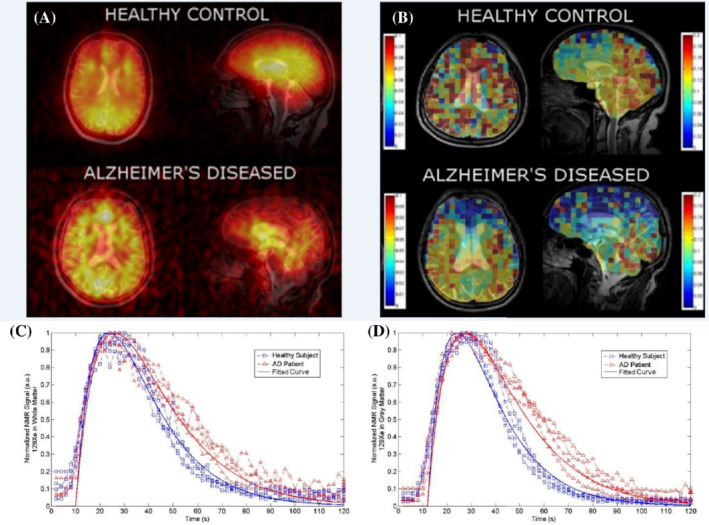
(A) Axial and sagittal ^129^Xe MRI of healthy controls and Alzheimer's disease (AD) participants. (B) Xenon washout parameter maps of healthy controls age‐matched to AD patients overlaid onto T_2_‐weighted anatomical images. MRS SNR of ^129^Xe‐WM (C) and ^129^Xe‐GM (D) spectral peaks as a function of time for healthy controls (blue) and AD participants (red). The participants inhaled 500 ml of HP ^129^Xe and held their breath for 20 s. ^129^Xe MRS from the brain region was acquired every 2 s. An increase in ^129^Xe signal after approximately 10 s was noticed as the ^129^Xe reached the brain. At 20 s, the participant exhaled and the ^129^Xe signal began to decrease at different rates for AD participants compared with healthy controls for white matter and gray matter. Images are reprinted with permission from the publisher^54^

Another neurological disorder that affects the cerebral blood flow and the brain tissues is Alzheimer's disease (AD). To investigate the possibility of using HP ^129^Xe imaging for AD detection, Hane et al. conducted an HP ^129^Xe washout study in 2018.^54^ Four participants diagnosed with mild to moderate AD and 4 age‐matched healthy volunteers underwent HP ^129^Xe gas MRS and MRI during a 20‐s breath‐hold. Sixty dynamic MRS scans were acquired every 2 s starting from initialization of the breath‐hold. Three dynamic balanced SSFP MRI images were acquired at 10, 20, and 30 s after gas inhalation. Five different peaks were observed using MRS that agreed with the spectroscopy results from Rao et al. in 2016.^42^ Interestingly, however, in this study, the ^129^Xe signal from gray matter was 43% lower in AD participants compared with healthy volunteers, and the white‐matter peaks were not statistically different between the two subject cohorts. This reduction in HP ^129^Xe signal resulted in a decrease in the SNR of images acquired from the AD subjects (Figure 12A). The white‐matter and gray‐matter spectral peaks were monitored over time: The ^129^Xe washout half‐life for healthy participants was 20 and 16 s for white matter and gray matter, respectively, whereas the ^129^Xe washout half‐life for participants with AD was 42 and 43 s in white matter (Figure 12C) and gray matter (Figure 12D), respectively. The analysis of the dynamic ^129^Xe MR images (Figure 12B) revealed that the Xe washout parameters were similar in the caudal brain regions for both cohorts of participants, whereas the prefrontal regions showed a reduction of the localized ^129^Xe washout parameter in AD volunteers. Therefore, a ^129^Xe retention parameter was proposed as a potential biomarker for AD detection.

## 9 DISCUSSION

HP ^129^Xe MRI of the brain is a promising medical imaging modality that is currently under extensive development. Thirteen articles on HP ^129^Xe brain imaging were published between the period of the invention of HP ^129^Xe MRI in 1994, and 2008, while 26 articles were published between 2008 to the present. Furthermore, the number of papers published in the HP^129^Xe brain imaging field grew steadily over the past decade. The most prominent practical application of HP ^129^Xe brain imaging so far has been its use for cerebral perfusion imaging.^52^, ^54^, ^68^, ^72^ The free dissolution of HP ^129^Xe in the pulmonary blood renders ^129^Xe an exogeneous blood‐flow contrast agent. The signal intensity of HP ^129^Xe brain images is determined primarily by the tissue perfusion, but is further regulated by the level of polarization, the amount of Xe that is inhaled, and the concentration of xenon that is transferred to the brain (Xe solubility is 0.17 in the blood, 0.135 in gray matter, and 0.224 in white matter^48^). Furthermore, HP ^129^Xe is an exogeneous perfusion inhalation contrast agent that does not provide any undesired background signal.

Unlike ^1^H‐ASL perfusion imaging, the lack of background signal and need for intensive signal averaging both provide some of the main advantages of HP ^129^Xe brain perfusion imaging. With this in mind, the acquisition protocol for HP ^129^Xe MRI can potentially be simpler compared with that required for ASL MRI for future implementation in the clinic. Because no signal averaging is required, the specific absorption rate of HP ^129^Xe perfusion imaging scans can also potentially be lower compared with ASL proton scans. Also, in contrast to ASL MRI, HP ^129^Xe brain perfusion imaging can be performed at low field, due to both the exogeneous nature of HP ^129^Xe and the fact that its signal has a weak dependence of the Bo magnetic field strength.^73^, ^74^, ^75^ While ASL perfusion imaging is already well developed for clinical use, HP ^129^Xe perfusion imaging is still in its infancy; further improvements to its method of signal acquisition, and increases to the ^129^Xe polarization level, will be required to render HP ^129^Xe perfusion imaging competitive with ASL MRI.

Another advantage of HP ^129^Xe brain perfusion imaging is its ability for extremely rapid image acquisition. This fact originates from the nonrecoverable nature of the hyperpolarized longitudinal magnetization. Because the HP state is a metastable non‐equilibrium state, spin–lattice relaxation destroys the longitudinal component of its net magnetization over time. Therefore, the use of a short TR is highly beneficial, which also results in a short scan time. HP ^129^Xe brain image acquisition times are typically on the order of seconds^54^, ^72^ (Table 2), although further shortening of the scan time is usually possible. Such short image‐acquisition times also reduce the sensitivity of HP ^129^Xe cerebral perfusion imaging to motion artifacts. In contrast, ASL perfusion imaging scans usually require several minutes due to the need for multiple signal averages, which makes conventional ASL MRI techniques very sensitive to motion artifacts.^76^, ^77^, ^78^

**TABLE 2:** Image parameter comparison for human HP ^129^Xe perfusion imaging, ^1^H ASL perfusion imaging, and HP ^129^Xe structural imaging

Imaging parameter	HP ^129^Xe perfusion imaging	^1^H ASL perfusion imaging	HP ^129^Xe structural imaging
SNR (arb. units)	26 ± 4.36^68^; 11.2 ± 2.9 (sagittal)^52^; 9.5 ± 2.9 (axial)^52^; 12.15 ± 5.45^70^	8.74 ± 2.02 (pCASL)^89^ 16.5 ± 2.2 (pCASL)^90^ 21.5 ± 3.6 (VSASL)^90^ 30.7 ± 10.1 (CASPR)^91^	18.76 ± 4.95 (axial)^60^ 19.47 ± 3.25 (sagittal)^60^;
Acquisition matrix	32 × 32^67^, ^70^, ^72^; 20 × 20^52^	64 × 64^89^, ^90^, ^92^, ^93^, ^94^, ^95^, ^96^; 73 × 73^91^ 128 × 128^97^	32 × 32^60^
Reconstruction matrix	80 × 80^67^, ^72^; 48 × 48^67^; 32 × 32^52^, ^70^;	64 × 64^89^, ^94^, ^96^; 128 × 128^90^, ^98^	32 × 32^60^
Number of slices	1^52^, ^67^, ^70^, ^72^	1^89^; 8^90^; 50^91^	5^60^
Slice thickness (mm)	50^67^, ^72^; 70 (axial)^52^; 130 (sagittal)^52^;	3^91^ 4^90^; 128^89^	20^60^
TR (ms)	34^67^, ^72^; 6.1^52^, ^70^	4000–4600^89^, ^90^, ^95^, ^98^ 6300^91^	6.2^60^

Abbreviations: CASPR, Cartesian acquisition with spiral profile reordering; pCASL, pseudo‐continuous ASL; VSASL, velocity‐selective ASL.

Despite the aforementioned advantages of HP ^129^Xe cerebral perfusion imaging, this methodology currently has several limitations. First, to perform HP ^129^Xe MRI, the research center or clinical site must possess an MRI scanner capable of performing multinuclear imaging. In addition, a high‐yield ^129^Xe polarizer (an expensive piece of equipment) is needed, in addition to dedicated MRI coils tuned to the resonance frequency of ^129^Xe. It is desirable to use a dual‐tuned ^1^H/^129^Xe RF head coil, as initial ^1^H brain localization is required before HP ^129^Xe brain imaging. Additionally, the use of isotopically enriched ^129^Xe is often required to achieve acceptable SNR levels, as the concentration of HP ^129^Xe is relatively low in the brain tissues. The necessity of specialized equipment and isotopically enriched ^129^Xe gas renders HP ^129^Xe brain perfusion imaging much more expensive than conventional clinical ^1^H perfusion MRI techniques.

As previously mentioned, another challenge for HP ^129^Xe brain imaging is the relatively low concentration of ^129^Xe dissolved in brain tissues (on the order of μM^48^). Therefore, the overall HP ^129^Xe signal level originating from the brain is quite low, resulting in relatively low image SNR, which significantly limits the use of HP ^129^Xe for anatomical brain imaging. In common practice, to optimize the HP ^129^Xe brain image SNR, the acquisition matrix is typically kept at a low resolution, and a single slice image is commonly acquired. The most commonly used acquisition matrix is 32 × 32, which is two times smaller compared with the most frequently used acquisition matrix for ASL imaging. In addition, ASL‐based perfusion imaging techniques can acquire images with a slice thickness about 3–4 mm, whereas the minimum slice thickness achieved so far for HP ^129^Xe imaging is 20 mm. This yields an HP ^129^Xe voxel size that is at least 20 times larger compared with typical ASL voxel sizes. Recent advances in 3D‐GRE HP ^129^Xe brain imaging can help to increase the spatial resolution of HP ^129^Xe cerebral perfusion images and potentially render them comparable to modern clinical ASL standards. Further increases in the HP ^129^Xe polarization (ideally up to the theoretical limit of 86%^79^) could potentially facilitate the enhancement in the signal required for use of an acquisition matrix of 64 × 64, which would meet current clinical standards for perfusion imaging. Even if this is accomplished, a 64 × 64 acquisition matrix will not be sufficient for structural brain imaging with HP ^129^Xe, as conventional ^1^H MRI is capable of much higher resolution. Therefore, it can be foreseen that HP ^129^Xe brain MRI does not bode well as a new anatomical MRI imaging modality, but has great potential for applications in the fields of functional imaging, such as perfusion imaging, blood flow detection, and BBB permeability imaging.

Extensive development of both hardware and MR pulse sequences is required to increase the SNR and spatial resolution of HP ^129^Xe brain MRI. The highest reported level of ^129^Xe polarization used for brain imaging so far was about 50%,^52^, ^60^, ^70^ which is a significant advancement compared with polarization values used for earlier experiments.^40^, ^48^ An increase in the polarization level will produce a linear increase in the ^129^Xe signal level. In addition, an increase in the isotopic enrichment of ^129^Xe gas used for imaging will also give a linear increase in the image SNR. Finally, the development and implementation of a multichannel phased‐array receiver RF coil could also increase the SNR of HP ^129^Xe brain imaging. Although preliminary results have been reported using a six‐channel phased array ^129^Xe brain coil for in vivo single‐voxel spectroscopy,^80^ further implementation of the parallel imaging approach for imaging purposes is essential to advance HP ^129^Xe imaging of the human brain.

Alongside hardware development, future work must also be focused on imaging pulse sequence development and breathing protocol optimization. Due to the short TR requirements, HP ^129^Xe brain imaging mostly uses GRE imaging pulse sequences. Until recently, the most commonly used MR protocol for ^129^Xe brain imaging was a thick single‐slice 2D GRE image acquisition with standard sequential k‐space filling.^52^, ^54^, ^68^, ^72^ The use of non‐Cartesian k‐space trajectories, which oversample the center of a k‐space (such as radial trajectories) can further increase the image SNR, and may allow the acquisition of thinner slices and higher spatial resolution in HP ^129^Xe brain images. The downside of using non‐Cartesian k‐space trajectories is that they undersample the outer edges of k‐space, which results in blurriness of the image.

Recent development by Rao et al. allowed progression to 3D multislice isotropic ^129^Xe brain MRI spectroscopic imaging, which could be further implemented for brain oxygenation mapping and for voxel‐wise quantification of HP ^129^Xe dissolved in different brain compartments.^59^

Another pulse‐sequence approach worth pursuing is translation from 2D‐GRE to 3D‐GRE imaging. 3D‐GRE imaging will allow the image SNR to increase through additional phase encoding in the slice‐selection direction and will allow multislice image acquisition. A proof‐of‐concept demonstration of 3D‐GRE HP ^129^Xe imaging in humans was recently demonstrated by Grynko et al.^60^ In this study, use of a 3D‐GRE sequence allowed reduction of the voxel size by 93% compared with the 2D‐GRE imaging approach. Because all of the spins in the volume of interest get excited simultaneously, 3D‐GRE multislice imaging better uses the hyperpolarized magnetization, compared with 2D‐GRE multislice imaging. Combining 3D‐GRE pulse sequences with non‐Cartesian k‐space trajectories has the potential to further improve the quality of HP ^129^Xe brain images.

The breathing protocol is another vital factor that should be carefully considered. The most commonly used breathing protocol for HP ^129^Xe human brain imaging is the inhalation of 1 L of HP gas followed by a subsequent breath‐hold.^52^, ^54^, ^68^, ^72^, ^81^, ^82^ This approach, however, is associated with a high level of signal variability (∼30%).^70^, ^82^ This signal variability can be caused by numerous factors, such as the exact quantity of HP ^129^Xe gas dispensed in the bag each time, the T_1_ relaxation during gas storage before being administered, different concentrations of HP ^129^Xe in the lungs, cerebral perfusion values, lung–brain arterial transit times, and the amount of time into the breath‐hold when image acquisition begins. All of these factors affect the concentration of HP ^129^Xe dissolved in the brain at a particular moment in time. A recent study demonstrated that the use of a time‐resolved initial depolarization pulse (TOF technique) reduces the variability of the HP ^129^Xe signal by up to 2.4 times.^70^ Use of an initial depolarization pulse, therefore, is highly beneficial for all further HP ^129^Xe brain imaging studies. Despite achieving a significant reduction, however, the presence of an initial depolarization pulse did not completely eliminate the interbreath‐hold signal variability issue. A contributing factor that cannot be eliminated originates from variations in blood flow in the cerebral arteries feeding the tissues, which directly affects the HP ^129^Xe brain signal. The highest level of signal variability was observed to correspond to the brain region supplied by the posterior cerebral artery, whereas the lowest variability corresponded to the region supplied by the anterior cerebral artery.^70^

To maximize the image SNR, data acquisition should be performed once the brain tissues are saturated with HP ^129^Xe.^70^ Based on the various HP ^129^Xe brain uptake models previously developed,^48^, ^52^ the concentration of HP ^129^Xe in the brain reaches a maximum at approximately 15 s into the breath‐hold for typical values of cerebral perfusion, arterial transit times, and T_1_ relaxation times in the blood and brain tissues. To maximize the HP ^129^Xe SNR, therefore, the image should be acquired at this moment in time. If a subject cannot hold his or her breath for this amount of time (eg, subjects with pulmonary disorders or children), however, or if the imaging purpose is to acquire dynamic images over a breath‐hold (eg, to quantify HP ^129^Xe uptake), the resulting image SNR can potentially be lower.

It is worth considering other breathing protocols. One that might be of interest for future studies, and that might overcome some of these issues, is continuous breathing using HP ^129^Xe premixed with oxygen. Continuous breathing protocols have been used for animal lung studies,^83^, ^84^ and human lung studies with ^3^He^85^, ^86^, ^87^ and ^129^Xe.^88^ They might be beneficial for HP ^129^Xe brain imaging, as they can prolong the plateau of the maximum brain ^129^Xe concentration period. This could allow the conductance of longer scans, signal averaging, and acquisition of HP ^129^Xe brain images in subjects that are not capable of performing a long breath‐hold.

Currently, the main advantages of ASL over HP ^129^Xe cerebral perfusion imaging is the higher in‐plane spatial resolution (typically 64 × 64), thinner slices, and commercially available software for image analysis. As indicated in Table 2, the SNR values of HP ^129^Xe perfusion images are comparable with clinically available pseudo‐continuous ASL perfusion images. The recent implementation of a 3D‐GRE readout has further increased the SNR of HP ^129^Xe brain images up to the level that is comparable with velocity‐selective ASL. The spatial resolution of HP ^129^Xe images, however, remains at least four times lower compared with ASL techniques. To bridge this gap, further improvements to the HP ^129^Xe SNR, as discussed previously, can be converted into increasing the acquisition matrix and reducing the slice thickness.

In addition, the further development of mathematical models for HP ^129^Xe signal dynamics and its conversion into computer algorithms for HP ^129^Xe cerebral perfusion image calculation can improve the accuracy of HP ^129^Xe perfusion imaging. Additional experimental characterization of HP ^129^Xe in the brain should accompany the optimization of mathematical models, as there still remain multiple fundamental physical properties of HP ^129^Xe dissolved in cerebral blood and brain tissues that remain unknown. For example, accurate measurements of the T_1_ relaxation times of each of the HP ^129^Xe spectral components, as well as HP ^129^Xe diffusion coefficients, are required. Without these experimental data, it will not be possible to quantify cerebral perfusion accurately.

In spite of these shortcomings, it is inspiring that HP ^129^Xe brain imaging has already demonstrated its potential to image subjects with AD^54^ and stroke.^68^ Moreover, because the HP ^129^Xe brain signal depends on the cerebral perfusion, as well as the permeability of the BBB, it might also be useful for the detection of other diseases associated with cerebral blood flow changes (eg, Parkinson's disease,^99^ atherosclerosis^100^) or those with associated BBB impairment (eg, cerebral small vessel disease,^101^ multiple sclerosis^102^). Additionally, due to the high lipophilicity of xenon, HP ^129^Xe imaging may also be useful for brain cancer detection. Although there were two preliminary studies on this application,^103^, ^104^ the validation of HP ^129^Xe brain imaging for use in cancer detection will require further proof‐of‐concept studies.

## 10 CONCLUSIONS

HP ^129^Xe brain imaging is a promising imaging modality that has been developing rapidly over the past several years. With further development, it has the potential to provide rapid and direct imaging of perfusion with an SNR comparable to that of ASL perfusion imaging, even at low field. HP ^129^Xe perfusion imaging has an extremely fast acquisition time (less than 20 s), has no endogenous background signal, and is much simpler in practice than other MRI techniques from the MR pulse‐sequence design point of view. The rapid acquisition times possible for HP ^129^Xe perfusion images ensure its insensitivity to motion artifacts. In addition, due to xenon's ability to cross the BBB, assessment of BBB permeability can readily be performed using HP ^129^Xe MRI.^53^, ^58^ HP ^129^Xe perfusion imaging has the potential to become a valuable new perfusion imaging technique that eventually will take its place alongside that of clinical ASL MRI and dynamic contrast‐enhanced perfusion imaging.
